# Assessing Parent–Infant Bonding in a Community Perinatal Mental Health Service

**DOI:** 10.1002/cpp.70036

**Published:** 2025-03-05

**Authors:** Grace Baptie, Karen Yirmiya, Camilla Rosan, Cathy Coombs, Kim Alyousefi‐van Dijk

**Affiliations:** ^1^ Clinical, Health and Educational Psychology University College London (UCL) London UK; ^2^ Early Years and Prevention Anna Freud Centre for Children and Families London UK; ^3^ Perinatal Services Birmingham and Solihull Mental Health NHS Foundation Trust Birmingham UK

**Keywords:** CORE‐10, parent–infant bond, perinatal mental health, Postpartum Bonding Questionnaire, screening measures

## Abstract

**Objectives:**

Perinatal mental health (PMH) services support the mental health needs of women and birthing people in pregnancy and postnatal, alongside the developing relationship between parent and infant. Mental health symptoms in PMH services are routinely screened for, yet there are inconsistencies in whether parent–infant bond is assessed and how. The aim of this study is to assess the predictive validity of screening for parent–infant bonding difficulties (Postpartum Bonding Questionnaire (PBQ)) and psychopathology (CORE‐10) to predict patient outcomes at discharge from a PMH service.

**Methods:**

Secondary analysis of clinical data from a PMH service in Birmingham, United Kingdom, encompassed 948 patient records. A structural equation model was constructed on patient data containing PBQ and CORE‐10 scores recorded at initial assessment and discharge from the service.

**Results:**

Analysis revealed a significant decrease in bonding difficulties and psychopathology scores from initial assessment to discharge from the service. The predictive model showed CORE‐10 scores at discharge were predicted by both initial CORE‐10 and PBQ scores, whereas PBQ scores at discharge were predicted solely by initial PBQ scores. Demographic factors including age, parity and ethnicity did not present any direct association with psychopathology or bonding difficulties at either timepoint.

**Conclusions:**

This analysis provides evidence of a pathway between early parent–infant bond and later psychopathology symptoms, which exists independently from the pathway between psychopathology symptoms at intake and discharge alone. These findings support embedding self‐report assessments of parent–infant bond, in addition to measures of psychopathology, to better predict patient outcomes at discharge from a PMH service.

**Summary**

- Self‐report measures of parent–infant bond are useful tools for clinical assessment and intervention in identifying and responding to perceived difficulties in the parent–infant relationship.
- Initial assessment of parent–infant bonding, using a self‐report measure, improves predictive validity of patient outcomes at discharge from a community PMH service.
- Initial assessment of maternal psychopathology alone does not predict later parent–infant bonding difficulties.
- Consideration of clinical thresholds for impaired parent–infant bond using the PBQ and cultural sensitivity of items relating to infant‐focused anxiety are required.

## 1 Introduction

Perinatal mental health (PMH) problems are a global public health issue. To improve PMH support, there is an essential need for holistic perinatal care that centres the parent–infant relationship alongside the mother or birthing person's mental health needs. The parent–infant bond is dynamic and bidirectional, characterised by nurturing behaviours from the parent and corresponding responses from the infant that stimulate further caregiving. Typically, bonding challenges are viewed from the parent's perspective, focusing on their feelings towards the infant, rather than observable relationship or attachment difficulties. For the purposes of this study, the bond is described as a representation of a parent's emotional response to their infant and is used to describe parent's internal, affective experiences towards their child (Brockington et al. 2001). Research indicates that bonding difficulties after birth are usually transient (Robson and Kumar 1980), but for some, impaired bonding is shrouded in shame and guilt and can manifest as emotional ambivalence or anger and, in extreme cases, an increased risk of neglect, abuse or emotional rejection (Brockington 2004). The infant's relational context is a critical feature of their social emotional health, and impaired parent–infant bond is associated with negative developmental outcomes for the child (Le Bas et al. 2022). Current UK policy initiatives are driving the expansion of services aimed at supporting families to develop positive parent–infant relationships (Department of Health and Social Care and Department for Education 2022), yet identifying effective measures to assess the parent–infant bond remains challenging (Olander et al. 2021).

The absence of standardised diagnostic criteria to define the presence or severity of difficulties in the parent–infant bond leads to significant variation in how impairments are defined, assessed and measured across different services and studies (O'Dea et al. 2023). As a result, prevalence rates of bonding difficulties are difficult to estimate and reliable identification of parents in need of support with their relationship with their infant remains patchy. Observations of parent–infant interactions are routinely employed for screening outcome assessments and within therapeutic interventions addressing relationship difficulties. Various frameworks exist for coding interaction observations, effectively assessing attachment behaviours and parental sensitivity. However, these methods do not capture the parents' internal representation of their bond with their infant. Consequently, national guidelines for PMH services recommend incorporating both observational measures and self‐report measures to assess parent–infant relationship difficulties (Royal College of Psychiatrists 2018). A review of 17 self‐report measures of parent–infant bond found most measures to be acceptable in terms of clinical application, but the review identified a lack of evidence for robust psychometric properties across measures. Only one measure, the Postpartum Bonding Questionnaire (PBQ) (Brockington et al. 2001), demonstrated sufficient structural validity, internal consistency and reliability from high‐quality evidence (Wittkowski et al. 2020).

### 1.1 PBQ

. The PBQ was devised in 2001 as a tool to assess a mother's emotional response to her infant (Brockington et al. 2001). The PBQ is a 25‐item self‐report measure and consists of four subscales which reflect impaired bonding, rejection and anger, anxiety about care and risk of abuse. Items are rated by parents on a 6‐point Likert scale from ‘*always*’ to ‘*never*’ and include ‘I feel close to my baby’ and ‘I feel trapped as a mother’. Originally developed and validated in the United Kingdom, the PBQ has since been translated into at least 10 languages and is widely used in research and clinical practice worldwide (e.g., Suetsugu et al. 2015; Vengadavaradan et al. 2019). Although the PBQ has seen multiple adaptations and translations, there remains a significant lack of cross‐cultural validation for parent–infant relationship measures, as well as a lack of confirmation or replication of the four‐factor structure (Wittkowski et al. 2020). Nevertheless, the PBQ is widely adopted and is the recommended self‐report measure in the *Framework for Routine Outcome Measures for Community Perinatal Mental Health Services* (Royal College of Psychiatrists 2018).

### 1.2 Community PMH Services

Since development of the PBQ, UK PMH policy and practice have seen significant changes. With increased national investment in prevention and support during the first 1001 days (Department of Health and Social Care 2021), community PMH services now provide more expansive, multidisciplinary support for parents with wider and more diverse needs during the perinatal period, with a key focus on the parent–infant relationship. Interventions for bonding difficulties are qualitatively different from interventions for maternal mental health symptoms such as anxiety or depression. Cognitive, behavioural and pharmacological approaches can help alleviate mental health symptoms, but they are not effective in improving parent–infant bond (O'Mahen et al. 2013), whereas interventions for bonding difficulties primarily focus on improving parental sensitivity and reflective functioning and have been shown to benefit both parents' psychological wellbeing and their relationship with their baby (Fonagy, Sleed, and Baradon 2016).

Bonding difficulties are more prevalent amongst parents receiving PMH care, but there remains a substantial proportion of parents who experience mental health difficulties postpartum but do not present with bonding difficulties (O'Dea et al. 2023). Bonding difficulties can also pose as a risk factor for heightened anxiety and symptoms associated with depression such as guilt, shame and anhedonia (Beato et al. 2022). As such, mental health difficulties and difficulties in parent–infant bond share a bidirectional overlap but represent distinct constructs that do not always co‐occur. In practice, it is important to incorporate assessment tools that specifically focus on the parent–infant bond for reliable identification of parents in need of relational support, rather than rely on measures of mental wellbeing alone. The PBQ is a promising screening tool for bonding difficulties to form part of a comprehensive assessment in community PMH services, but there is wide variability and a lack of consensus in practice as to which outcome measures should, and are, being used to assess the parent–infant relationship (Centre for Early Childhood Development 2023).

### 1.3 Aims and Hypotheses

Given the changing landscape of PMH support and the impetus in political agendas focused on the parent–infant relationship, the current study analyses a large dataset from a community PMH service in Birmingham, United Kingdom, incorporating both the PBQ and mental health screening measures. The aim of this analysis is to assess the predictive validity of screening within an initial assessment at a community PMH service for parent–infant bonding difficulties using the PBQ, in addition to maternal psychopathology measured by the CORE‐10, to predict patient psychological outcomes at discharge from the service. We hypothesise that bonding difficulties will show some correlation with psychopathology given the established overlap between mental health symptoms and parent–infant bond, and both will present a decrease from initial assessment to assessments taken at discharge from the service. The exploratory hypothesis tests whether PBQ scores at intake will predict CORE‐10 scores at discharge independently of initial CORE‐10 scores and, conversely, whether CORE‐10 scores at intake will predict PBQ scores at discharge beyond initial bonding scores.

## 2 Method

### 2.1 Sample

The current study is a secondary analysis of clinical data retrieved from a community PMH service in Birmingham, United Kingdom.^1^ Data was collected between January 2019 and January 2022 as part of routine, universal screening assessments for patients in the service. The sample included 948 patients, who were aged between 18 and 48 years old (*M* = 29.64 years old, SD = 5.77). Full sociodemographic characteristics of the study cohort are presented in Table 1.

**TABLE 1:** Sample characteristics from sociodemographic frequencies and means, diagnoses and mental health and bonding difficulties at initial assessment and discharge from the service.

	*N* (%)	*M*	SD	Range
Age (y) (*N* = 924)		29.64	5.77	18–48
Number of children (*N* = 808)		2.01	1.14	0–6
Ethnicity (*N* = 770)				
White	464 (50.2%)			
Asian	175 (18.9%)			
Black	48 (5.2%)			
Mixed	46 (5%)			
Other	21 (2.3%)			
No ethnicity recorded	170 (18.4%)			
ICD‐10 diagnoses (*N* = 167)				
No diagnosis recorded	716 (77.5%)			
Anxiety disorders	29 (3.1%)			
Mood disorders	72 (7.8%)			
Psychotic‐related disorders	28 (3%)			
Personality and other disorders	38 (4.1%)			
CORE‐10 initial assessment (0–40)	924	15.83	8.93	0–38
CORE‐10 discharge (0–40)	572	12.32	9.67	0–36
PBQ initial assessment (0–125)	925	12.22	13.83	0–86
PBQ discharge (0–125)	266	10.93	12.93	0–85

### 2.2 Service Context

The clinical data is from a community PMH service in the Midlands which delivers PMH services for women and birthing people, with moderate to severe/complex mental health difficulties (and their partners and babies) from pregnancy through to 2 years postpartum to a diverse, urban community. The service is run by a multidisciplinary team including psychiatrists, psychological therapists, psychiatric nurses, occupational therapists, social workers, nursery nurses and peer support workers. When patients are referred and accepted to the service, they receive an initial assessment and will receive the following measures: CORE‐10, HoNOS and PBQ. Following assessment, patients are offered one or multiple types of support, such as care co‐ordination, psychological therapy (e.g., CBT, EMDR, anxiety management, emotional coping skills group), pharmacological intervention, occupational therapy intervention (e.g., sensory assessment, return to work support), psychosocial support and parent–infant intervention (e.g., Baby Massage, Circle of Security Parenting, Video Interaction Guidance, Watch Me Play, Five to Thrive).

### 2.3 Procedure

Clinical data was collected as part of routine assessments during initial patient assessment and discharge. Initial assessments were predominantly carried out by community PMH service NHS staff members and inputted onto a patient electronic record system. Approximately half of the patients (*n* = 398, 42%) also completed assessments of parent–infant bonding difficulties within 3 weeks from their initial mental health assessment. The average duration in the service was 34 weeks (SD = 23 weeks). A subsample of 578 patient records had an additional mental health assessment at discharge, and 268 of these patients were also assessed for parent–infant bonding difficulties within 3 weeks of their mental health discharge assessment, enabling analysis of symptom trajectory from initial assessment to discharge from the service for a subgroup of patients.

### 2.4 Measures

*Demographic variables* included maternal age, ethnicity and number of children.

#### 2.4.1 Mental Health Difficulties—Clinical Outcomes in Routine Evaluation‐10

The CORE‐10 is a 10‐item monitoring tool covering various mental health difficulties scored between 0 and 40 with higher scores indicative of greater distress (Barkham et al. 2013). The CORE‐10 has been shown to have good psychometric properties with good internal reliability (0.90) and is routinely used with people presenting common mental health difficulties in primary care settings.

#### 2.4.2 Parent–Infant Bonding Difficulties—PBQ

The PBQ is a 25‐item self‐administered measure designed to provide an early indication of disorders within parent–infant relationships through the assessment of parent's feelings and attitudes towards their infant (Brockington et al. 2001). The PBQ is frequently used in research and clinical practice and has demonstrated acceptable reliability and reasonable validity (Mathews et al., 2019). The data extracted contained PBQ total scores (0–125) as well as total scores for each of the four subscales that make up the questionnaire: ‘*general bonding difficulties*’, ‘*rejection and pathological anger*’, ‘*infant‐focused anxiety*’ and ‘*risk of abuse*’, with higher scores reflecting greater bonding difficulties.

#### 2.4.3 Psychiatric Diagnoses

A subsample of patient records (*n* = 167, 17.62%) also contained diagnosis data in the form of ICD‐10 diagnoses. Diagnoses were grouped into four categories: anxiety disorders, mood disorders, psychotic‐related disorders and personality/other disorders.

### 2.5 Statistical Analysis

Descriptive characteristics of the patients were summarised as frequencies, percentages and mean total scores with standard deviations (SDs). To describe our study population's CORE‐10 scores, interpretations were based on guidelines from Connell and Barkham (2007) and PBQ total and subscale scores; we utilised criteria from the original validation by Brockington, Fraser and Wilson (2006), which provides validated guidelines for interpreting PBQ scores. Changes in CORE‐10 and PBQ scores from initial assessment to discharge were examined using paired samples *t*‐tests and McNemar's tests. Pearson correlations explored the relationships between maternal age, number of children, CORE‐10 and PBQ scores. A structural equation model (SEM) was fitted to examine associations between demographic variables and scores at intake and discharge. To assess model fit, the following indices were employed: *χ* ^2^, comparative fit index (CFI), Bollen's incremental fit index (IFI) and the root mean square error of approximation (RMSEA). CFI and IFI values ≥ 0.90 and RMSEA values ≤ 0.08 are considered to indicate a good fit (Hu and Bentler 1999). Analyses were performed using SPSS (Version 25) and the Lavaan package for RStudio (Version 2023.06.0 + 421), with significance level set at 0.05.

## 3 Results

### 3.1 Descriptives at Initial Assessment

PBQ results from initial assessment in the service indicated most patients (*n* = 767, 84.8%) scored below the threshold considered to indicate impaired parent–infant bond (25 or less), while a smaller portion (*n* = 135, 15.2%) scored over this threshold (26 or higher) indicating potential disorder in the parent–infant relationship. Similarly, most patients scored below the cut‐off on the first, general bonding difficulties’ subscale, with only 137 (15.15%) scoring 12 or above, which is the cut‐off for this factor. Meanwhile, only 12 (2.3%), 50 (5.5%) and 3 (0.3%) patients scored above the cut‐offs for the remaining three factors: rejection and anger, infant‐focused anxiety and risk of abuse, respectively. Regarding the CORE‐10 at initial assessment, 637 patients (69%) scored above the cut‐off of 11, which indicates psychological distress and is used to differentiate between clinical and nonclinical populations. Amongst those above the clinical cut‐off, 120 patients (13%) scored within the ‘mild distress’ range of 11–14, 162 (17.5%) within the ‘moderate distress’ range of 15–19, 173 (18.7%) within the ‘moderate‐to‐severe’ range of 20–24 and 182 (19.70%) scored within the ‘severe distress’ range of 25–40, indicating severe mental health difficulties.

### 3.2 Differences Between Initial Assessment and Discharge

**FIGURE 1:**
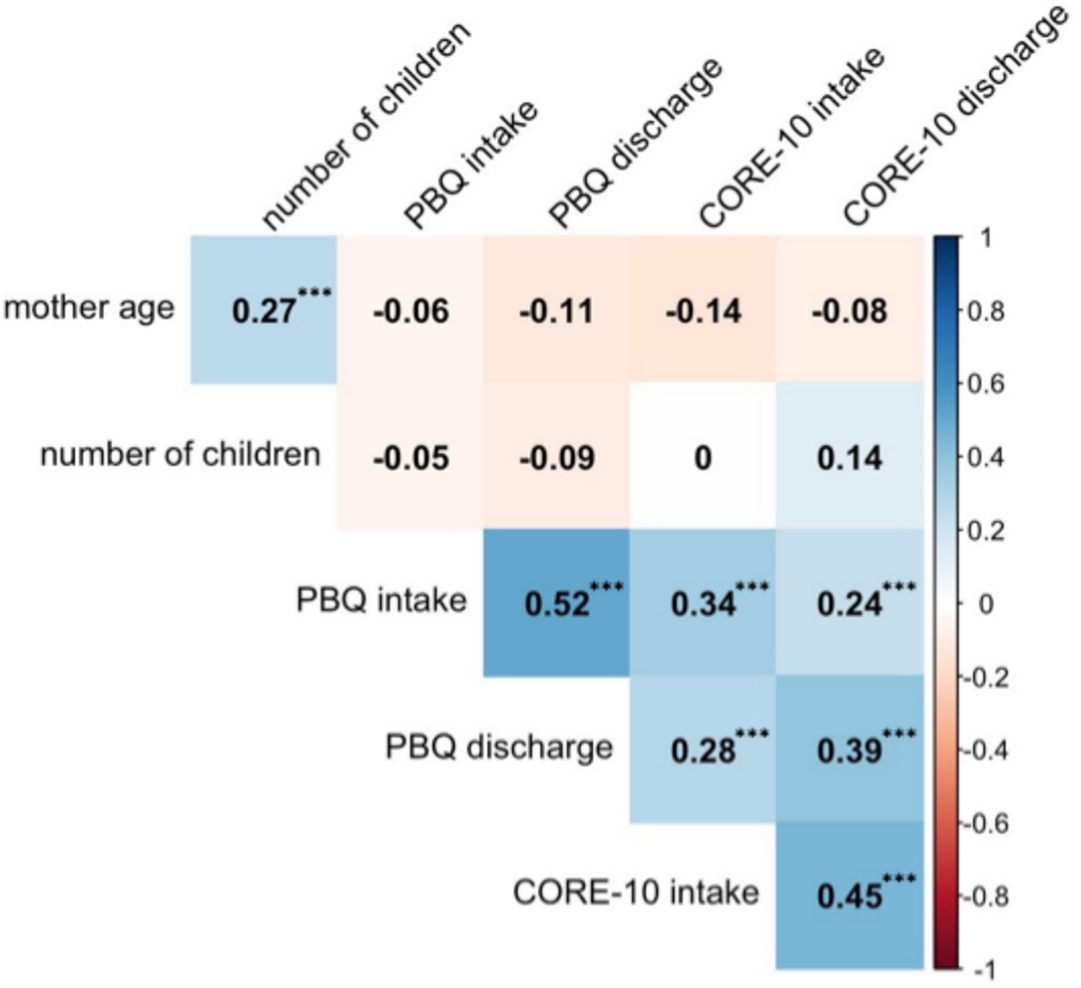
Pearson's R correlation heatmap of demographic variables and CORE and PBQ scores at initial assessment (intake) and discharge from the service.

Differences between initial assessments and discharge scores are presented in Figure 1. In line with our hypothesis, a paired sample *t*‐test revealed a significant decrease in average levels of psychopathology symptoms across the sample as measured by the CORE‐10, from *M* = 15.83 (SD = 8.93, *n* = 572), indicating ‘moderate’ psychological difficulties at intake, to *M* = 12.32 (SD = 9.68, *n* = 572) at discharge, which falls within the ‘mild’ psychological distress range, (*t*(571) = 9.25, *p* < 0.001). At discharge, 293 (51.2%) patients scored below the clinical cut‐off, 57 (10%) scored within the ‘mild distress’ range of 11–14, 73 (12.8%) scored within the ‘moderate distress’ range of 15–19, 69 (12.1%) scored within the ‘moderate‐to‐severe’ range of 20–24 and 80 (14%) scored within the ‘severe distress’ range of 25–40. Likewise, we found a statistically significant decrease in bonding difficulties over time as measured by the PBQ from initial assessment (*M* = 12.22, SD = 13.83, *n* = 925) to discharge (*M* = 10.93, SD = 12.93, *n* = 266) (*t*_(105)_ = 4.541, *p* < 0.001). Additionally, a significant change was observed in the proportion of patients scoring above the cut‐off of 26 or more on the total PBQ score. The proportion of patients scoring above the cut‐off significantly decreased from initial assessment (15%) to discharge (3%) (McNemar's *χ* ^2^ = 9.59, *p* = 0.002). Further analysis using paired *t*‐tests showed significant improvements across various subscales of the PBQ. For the general bonding difficulties’ scale, there was a significant decrease, *t*_(262)_ = 4.44, *p* < 0.001, with a mean difference of 2.03, indicating an improvement in the overall bonding measured. Similarly, scores for rejection and pathological anger showed significant reductions, *t*_(262)_ = 4.32, *p* < 0.001, with a mean difference of 1.34. Significant decreases were also found for the infant‐focused anxiety subscale, *t*_(262)_ = 5.30, *p* < 0.001, with a mean difference of 1.16. However, no significant difference was observed for the risk of abuse subscale, *t*_(262)_ = −0.29, *p* = 0.773, likely due to a floor effect.

### 3.3 Correlations

As predicted, a moderate, positive correlation was found between CORE and PBQ scores at initial assessment (*r* = 0.371, *p* < 0.001) and at discharge (*r* = 0.381, *p* < 0.001). A significant positive correlation was observed between the number of children and CORE‐10 scores at both initial assessment (*r* = 0.07, *p* = 0.044) and discharge (*r* = 0.144, *p* < 0.001), indicating that patients with more children tended to have higher CORE‐10 scores. In contrast, no significant correlation was found between the number of children and PBQ scores at initial assessment or discharge. Maternal age did not correlate with CORE or PBQ scores at any timepoint (see Figure 1).

### 3.4 Group Comparisons

Group comparisons indicated no significant differences between ethnic groups in total CORE‐10 and total PBQ scores at initial assessment or discharge. However, when examining the four subscales of the PBQ, multivariable analysis revealed a significant difference between different ethnic groups at initial assessment (*F*_(5)_ = 1.86, *p* = 0.020) but not at discharge (*F*_(5)_ = 1.42, *p* = 0.124). Post hoc analysis using the Bonferroni test revealed a significant difference in the infant‐focused anxiety subscale between White and Asian patients. Specifically, White patients reported higher infant‐focused anxiety than Asian patients at initial assessment (White *M* = 3.59, SD = 3.28; Asian *M* = 2.69, SD = 3.19; mean difference (*I* − *J*) = 0.90, *p* = 0.022). There were no differences across the other three subscales of the PBQ. No significant difference was found between the four diagnostic groups (anxiety disorders, mood disorders, psychotic‐related disorders and personality/other disorders such as eating disorders, somatoform disorder or adjustment disorder) in terms of PBQ scores at either timepoint.

### 3.5 Predictive Model

**FIGURE 2:**
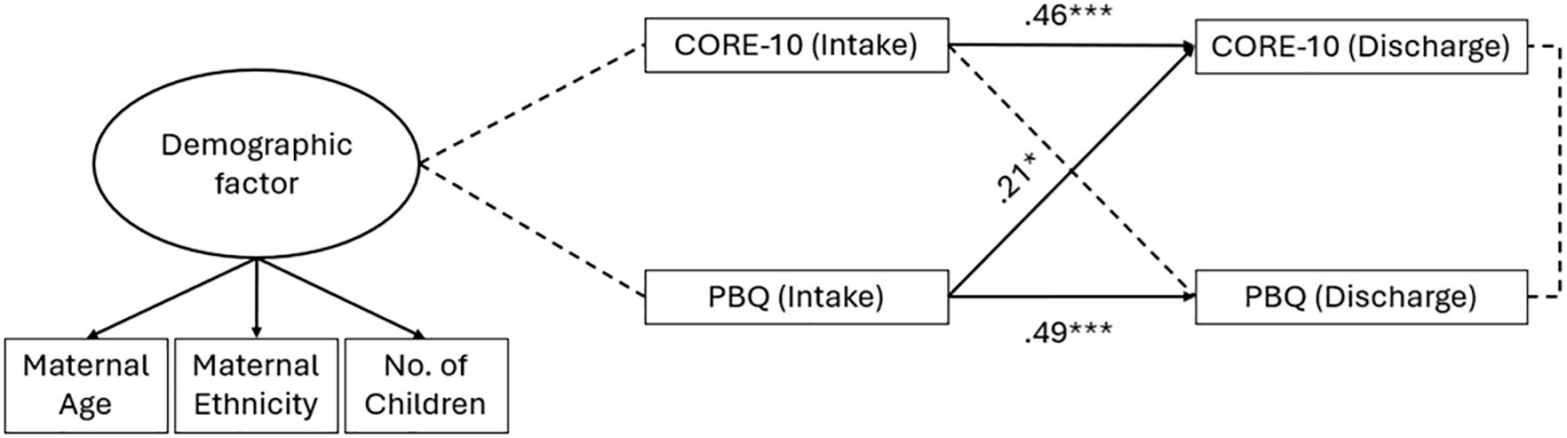
Structural equation model of CORE‐10 and PBQ scores at initial assessment (intake) and discharge from a community PMH service.

To address the primary research question, a SEM analysis was conducted to assess the predictive validity of screening for parent–infant bonding difficulties in addition to psychopathology symptoms at initial assessment in a community PMH service to predict patient outcomes at discharge from the service. A latent factor model incorporating demographic variables including maternal age, minoritized ethnicity (White vs. other) and number of children (0–6) was constructed to control for potential confounding effects of these demographic characteristics. The model examining CORE‐10 and PBQ total scores from the demographic index revealed no direct association between the demographic factors and CORE‐10 and PBQ scores at initial assessment or discharge. However, CORE‐10 scores at discharge were predicted by both initial CORE‐10 and PBQ scores, whereas PBQ scores at discharge were predicted solely by PBQ scores at intake (see Figure 2). The model demonstrated a good fit to the data (*N* = 178: *χ* ^2^_(10)_ = 11.65, *p* = 0.31, TLI = 0.98, CFI = 0.99, RMSEA = 0.03).

## 4 Discussion

This secondary analysis of clinical data reveals a predictive relationship between a parent's perceived bond with their baby at initial assessment in a community PMH service and their psychopathology symptoms and bond at discharge from the service. As expected, both maternal psychopathology symptom presentation and parent–infant bond improved over time from first to last assessment. Interestingly, the model fitted on this data provides evidence of a pathway between early parent–infant bond and later psychopathology, which exists independently from the pathway between psychopathology symptom scores at intake and discharge alone. The relationship suggests maternal outcomes at discharge from a PMH service are predicted by both her own mental health symptom presentation and her perceived bond with her infant at intake to the service. These findings support a relational model of PMH which acknowledges the parent–infant relationship as a critical feature and key determinant of maternal psychopathology (Sameroff, McDonough, and Rosenblum 2004; Mauthner 1998). The current study presents justification for community PMH services to embed the PBQ as an assessment of parent–infant bond, in addition to measures of maternal psychopathology, as a tool to better predict a parent's symptom trajectory during their time in a PMH service. These findings align with UK‐wide quality standards for community PMH services to embed self‐report screening measures for the parent–infant relationship as part of a comprehensive assessment at intake and discharge from the service (Royal College of Psychiatrists 2023).

Despite finding significant correlations between psychopathology symptoms and parent–infant bonding difficulties at both timepoints, the model fitted to this data revealed no predictive pathway between initial maternal psychopathology symptom score and parent–infant bond at discharge. This suggests that other factors play a role in predicting a parent's perception of their bond with their infant at time of discharge from a PMH service beyond initial psychopathology symptom scores. This finding supports the theoretical concept of maternal mental health and parent–infant bond as two overlapping but distinct constructs that should be screened independently to accurately locate where difficulties primarily reside, within or outside the parent–infant relationship (O'Dea et al. 2023). Longitudinal research with community samples has demonstrated a predictive association between maternal mental health during pregnancy and perceived bond postpartum (Rossen et al. 2016). The predictive model on this data suggests potentially different predictive pathways for clinical populations and/or for multiple timepoints postpartum.

The data retrieved for this analysis was representative of a community PMH service catering to a diverse UK city. Analysis of demographic data revealed differences between patients of different ethnicities in self‐reported parent–infant bonding difficulties assessed at initial assessment in the service. Although there were no differences seen in total PBQ scores, when analysed by subscale, Asian patients reported significantly less infant‐focused anxiety at initial assessment compared to White patients. The subscale of infant‐focused anxiety consists of four items including ‘*my baby makes me feel anxious*’ and *‘I am afraid of my baby’*. The difference between groups may be attributed to a lack of cultural sensitivity for items relating to infant‐focused anxiety. In the current study, there were no differences between groups in PBQ scores at discharge from the service, which may be indicative of barriers to disclose difficulties in the parent–infant relationship upon entry to a PMH service that dissipates over time as trust develops with the service (Webb et al. 2021). Barriers associated with stigma and mistrust of services have been found to disproportionately affect South Asian women and birthing people seeking PMH support in the United Kingdom (Smith et al. 2019; Conneely et al. 2023; Wittkowski et al. 2011); therefore, it is advisable for practitioners and researchers to scrutinise the cultural sensitivity of self‐report parent–infant measures as well as the rigidity of any clinical thresholds put in place for parents to be able to access support for bonding difficulties.

### 4.1 Clinical Implications

Widening clinical assessments to encompass bonding difficulties with self‐report measures such as the PBQ not only improves the predictive validity of maternal outcomes but also promotes a holistic model of care that recognises the parent–infant relationship as a central tenet of both maternal and infant mental health. This relational model is reflected in parent–infant intervention research where improvements have been found across both parental mental health and within the parent–infant relationship (Cucciniello and Melia 2024; Fonagy, Sleed, and Baradon 2016), in contrast to interventions specifically addressing maternal mental health alone which do not consistently demonstrate crossover benefits to the parent–infant relationship (Nylen et al. 2006). Greater focus on parent–infant relationship is also reflected in NICE guidelines for postnatal care (NICE 2021) and in political agendas to improve universal Start for Life services for families during the perinatal period (Department of Health and Social Care and Department for Education 2022). The PBQ can be a useful screening tool to identify appropriate interventions, as well as in ongoing assessment of support needs for parent–infant bonding difficulties.

Bonding disorders can manifest in different ways and embedding self‐report measures of parent–infant bond allows practitioners to gain insight into parents' subjective experience of their relationship with their child, assess the severity of relationship disorder and enable identification of appropriate support as well as change over time (Klier and Muzik 2004). When combined with observational assessments of parent–infant interactions, self‐report measure can provide a more holistic view of both the perceived and observed relationship between parent and infant. The current study provides support for use of the PBQ as a self‐report tool to measure parent–infant bonding to improve predictive validity of maternal outcomes at discharge from a PMH service. The PBQ can also be used to guide intervention type and delivery based on parents' need for support for their perceived bonding difficulties. In Birmingham community PMH service, the PBQ is used initially to identify parents experiencing bonding difficulties and then used routinely during and after parents access parent–infant relationship support to assess the effectiveness of the intervention on parent–infant bonding. Although the current dataset does not contain information regarding interventions, the overall reduction in bonding difficulties over time is likely reflective of the systematic screening and tracking of parent–infant bonding difficulties that supports appropriate intervention preference and delivery. Opening conversations around the parent–infant bond should be supported by sufficient training and supervision structures for staff, robust safeguarding pathways and appropriate access to timely support for parents (Department of Health and Social Care 2024).

### 4.2 Research Implications

The clinical data revealed low prevalence rates for disordered bonding when using the original PBQ thresholds for total PBQ score (26 or more) from an early validation of the measure (Brockington, Fraser, and Wilson 2006). Indeed, the mean PBQ score at initial assessment into the service in this clinical sample was similar to average scores found amongst a community perinatal population in the United Kingdom (Davies et al. 2021). The PBQ was developed and implemented over two decades ago within a specialist Perinatal Psychiatry Service (Brockington et al. 2001), but since then, community PMH services have evolved to cater to a broader population and a wider spectrum of needs and difficulties. This evolution suggests that the original PBQ threshold of 26 or more may be inadequate for accurately identifying parents in need of parent–infant relationship support in a broader community population, highlighting the need for further research to establish scoring criteria that better align with the current scope of community PMH services.

Group comparisons in the patient data retrieved for this analysis presented demographic differences in the PBQ subscale of ‘infant‐focused anxiety’ between White patients and Asian patients. Despite the PBQ having multiple adaptations and translated versions, there is a scarcity of research into the cultural validity of the measure (Wittkowski et al. 2020). Future research would benefit from exploring the cross‐cultural applicability of the PBQ to ensure it accurately reflects and assesses bonding difficulties across diverse populations.

## 5 Limitations

The data extracted for this analysis are patient records from a community PMH service in Birmingham, United Kingdom, and therefore contain all the hallmarks and limitations of a real‐world clinical dataset. For example, patient records are updated onto a shared system by a large multidisciplinary team and there are inconsistencies in the systematic use and upload of screening measure records. To apply methodological consistency to the data, we only included patient records with a PBQ score within 3 weeks of their first CORE‐10 assessment so that we could deduce that these measures were completed as part of an initial assessment with the service. However, in doing so, we reduced the study sample by approximately half. Assessment scores at discharge were also inconsistent, and many patient records contained one PBQ score only or a second PBQ score that did not fall into a 3‐week window with their final CORE‐10 score, and these records were also excluded from the model.

The PBQ data retrieved contained total scores and scores across the four subscales of the PBQ but not individual item scores, which prevented factor analyses of differences between ethnic groups. Given the differences in Asian and White patient's self‐report infant‐focused anxiety found in this analysis, group comparisons of differential item functioning of the PBQ would be a useful avenue for further cross‐cultural evaluation.

## 6 Conclusions

This secondary analysis of clinical data presents the PBQ as a useful screening tool which predicts a mother's perceived bond with her infant as well as her mental health symptoms at discharge from a PMH service. The findings support a relational model of PMH which acknowledges the parent–infant relationship as a critical feature of maternal mental health. A relational model does not deny the existence of mental health difficulties residing outside of this relationship; indeed our analysis presents an independent pathway between psychopathology symptom scores over time, but it acknowledges that they are embedded within and affected by the parent–infant relationship. Assessments for parent–infant bond can provide improved understanding of where difficulties reside and the developmental course of maternal psychopathology, which could lead to different approaches to intervention at different stages of care. The findings are in line with national quality standards for community PMH services (Royal College of Psychiatrists 2023) and recent policy agendas that promote implementing self‐report screening assessments for parent–infant relationships (DHSC and DfE 2022).

## Ethics Statement

Ethical approval was not required for this secondary analysis as all data was anonymised, collected as part of routine assessments and contained no personal identifying information.

## Conflicts of Interest

The authors declare no conflicts of interest.

## Data Availability

The data that support the findings of this study are available from the corresponding author upon reasonable request.

## Endnotes

^1^
The service holds historic significance to the assessment of parent–infant bonding as the PBQ was first developed and implemented within the Birmingham Perinatal Psychiatry service over two decades ago (Brockington et al. 2001).
